# SP1‐induced SNHG14 aggravates hypertrophic response in in vitro model of cardiac hypertrophy via up‐regulation of PCDH17

**DOI:** 10.1111/jcmm.15073

**Published:** 2020-05-21

**Authors:** Yadong Long, Lin Wang, Zhiqiang Li

**Affiliations:** ^1^ Department of Cardiovascular Surgery Xiangya Hospital Central South University Changsha China; ^2^ Cardiovascular Surgery II National Center for Children’s Health Beijing Children’s Hospital Capital Medical University Beijing China

**Keywords:** cardiac hypertrophy, ceRNA, PCDH17, SNHG14, SP1

## Abstract

Cardiac hypertrophy (CH) is a common cardiac disease and is closely associated with heart failure. Protocadherin 17 (PCDH17) was reported to aggravate myocardial infarction. Present study was designed to illustrate the impact of PCDH17 and the mechanism of PCDH17 expression regulation in CH. CH model in vivo and in vitro was established by transverse aortic constriction (TAC) and Ang‐II treatment. Hypertrophy was evaluated in PMC and H9c2 cells by examining cell surface area and hypertrophic markers. Results demonstrated that PCDH17 was up‐regulated in CH in vivo and in vitro. PCDH17 knock‐down alleviated hypertrophic response in Ang‐II‐induced cardiomyocytes. By means of ENCORI database and a series of mechanism assays, miR‐322‐5p and miR‐384‐5p were identified to interact with and inhibit PCDH17. Next, lncRNA SNHG14 (small nucleolar RNA host gene 14) was validated to sponge both miR‐322‐5p and miR‐384‐5p to elevate PCDH17 level. The subsequent rescue assays revealed that miR‐322‐5p and miR‐384‐5p restored SNHG14 depletion‐mediated suppression on hypertrophy in Ang‐II‐induced cardiomyocytes. Besides, Sp1 transcription factor (SP1) was verified as the transcription factor activating both SNHG14 and PCDH17. Both SNHG14 and PCDH17 reversed SP1 knock‐down‐mediated repression on hypertrophy in Ang‐II‐induced cardiomyocytes. Together, present study first uncovered ceRNA network of SNHG14/miR‐322‐5p/miR‐384‐5p/PCDH17 in Ang‐II‐induced cardiomyocytes.

## 1 INTRODUCTION

Cardiac hypertrophy (CH) is an adaption reaction to stress stimuli for normal cardiac function maintenance. However, constant CH is often accompanied with poor cardiac remodelling, which might cause heart failure and even sudden death.[^1^, ^2^, ^3^] Recently, molecular functions in various diseases have been gradually uncovered.[^4^, ^5^, ^6^, ^7^, ^8^] It is well acknowledged that peptide hormones, growth factors and non‐coding RNAs are vital mediators in CH progression.[^9^] The potential molecular mechanisms beneath CH are in need of further exploration.

Protocadherin 17 (PCDH17) is in the subgroup of cadherin superfamily.[^10^] PCDH17 has been linked to heart diseases by several studies. For instance, PCDH17 level was induced by lncRNA DCRF to aggravate autophagy and myocardial fibrosis in diabetic cardiomyopathy.[^11^] Also, PCDH17 is highly‐expressed in vascular smooth muscle cells of myocardial infarction.[^12^] However, whether PCDH17 affected CH and how its expression was altered and regulated in CH remain covered.

Long non‐coding RNAs (lncRNAs) have a length of more than 200 nucleotides and are incapable to code proteins. However, lncRNAs play crucial regulatory roles in various diseases, including CH. LncRNA PEG10 elevates ANP and BNP levels in cardiomyocytes depending on HOXA9 overexpression, aggravating CH.[^13^] LncRNA TUG1 depletion has anti‐hypertrophy effects in Ang‐II treated cardiomyocytes.[^14^] LncRNA XIST interference reduces hypertrophic effects in phenylephrine (PE)‐induced cardiomyocytes by interacting with miR‐101.[^15^] LncRNA CHRF promotes CH development through sequestering miR‐93 from Akt3.[^16^] LncRNA ANRIL depletion attenuates apoptosis of myocardial cells in acute myocardial infarction through IL‐33/ST2 pathway.[^17^] SNHG14 is supported as a carcinogen aggravating cancers such as diffuse large B cell lymphoma and hepatocellular carcinoma,[^18^, ^19^] but there are no reports about the link of SNHG14 with PCDH17 and CH.

MicroRNAs (miRNAs) are non‐coding RNAs with a length of 18‐22 nucleotides and are featured with high conservation. As extensively reported, miRNAs are involved in CH and heart failure. MiR‐200a‐3p directly targets WDR1 and serves as a positive regulator in CH.[^20^] MiR‐129‐5p decreases HMGB1 mRNA and protein levels and improves cardiac function in rats with chronic heart failure.[^21^] MiR‐150‐5p serves as a predictor of overt heart failure in patients with univentricular heart.[^22^]

LncRNAs and miRNAs are widely reported to get involved in the ceRNA (competitive endogenous RNA) pattern in which lncRNA acts as a sponge of miRNA to free mRNA from the inhibitory influence of miRNA. The ceRNA pathway in cardiac disorders is commonly reported. For instance, lncRNA boosts cell apoptosis of cardiomyocytes in rats with acute myocardial infarction through interacting with miR‐449 to up‐regulate NOTCH1 expression.[^23^] LncRNA Gpr19 facilitates cell apoptosis and oxidative stress in neonatal rat ventricular cardiomyocytes and promotes ischaemia‐reperfusion injury via miR‐324‐5p/Mtfr1 pathway.[^24^] LncRNA 2810403D21Rik/Mirf acts as a ceRNA of miR‐26a to facilitate ischaemic myocardial injury.[^25^] LncRNA HIF1A‐AS1 is essential for ventricular remodelling after myocardial ischaemia/reperfusion injury via sponging miR‐204 to modulate SOCS2 expression.[^26^] However, the ceRNA pattern formed by SNHG14 and PCDH17 in CH has never been elucidated.

Our study started from PCDH17 to explore the impact of PCDH17 and how PCDH17 expression was regulated in  Ang‐II‐treated cardiomyocytes.

## 2 MATERIALS AND METHODS

### 2.1 In vitro CH animal model

Adult C57BL/6 male mice weighing 20‐25 g were available from Vital River Laboratory Animal Company (Beijing, China) for animal study, with the approval from the Animal Care and Use Committee of the Xiangya Hospital, Central South University. In vitro CH animal model was constructed via operating TAC surgery. Mice were injected intraperitoneally with 100 mg/kg of ketamine and 10 mg/kg of xylazine, then transverse thoracic aorta was dissected. Afterwards, mice were placed at 37℃ on ventilator for 7 days, and echocardiography was applied to check the pressure gradient of mice in TAC group. The sham operation was conducted for the matched mice undergoing the same procedures to TAC group, except aorta seam.

### 2.2 Cell culture and treatment

After splitting the cardiomyocytes to small pieces, tissues were digested at 37℃ in HEPES‐buffered saline. Then, trypsin was neutralized with 10% calf serum; cells were centrifuged and suspended in DMM/F12 (Invitrogen, Carlsbad, CA) with the supplements. To extract primary cardiomyocytes (PCM), cells were seeded on collagen‐coated silicone sheet for 24 hours and then incubated in serum‐free medium for 24 hours. The rat cardiac cells H9c2 were available from the Chinese Academy of Sciences (Shanghai, China) for this study. In vitro CH cell model was acquired by treating PCM and H9c2 cells with 1 mmol/L of angiotensin II (Ang‐II, Sigma‐Aldrich, St. Louis, MO) for 48 hours.

### 2.3 Extraction of total RNAs and quantitative real‐time PCR (qRT‐PCR)

Total RNAs were extracted from cardiomyocytes in each group using TRIzol standard method (Invitrogen), reversely transcribed employing the 5X All‐In‐One RT MasterMix (abmGood, Vancouver, Canada). To assess gene expression, qRT‐PCR was conducted on ABI 7500 fast real‐time PCR system (Applied Biosystems, Foster City, CA), with GAPDH or U6 as normalized gene. Relative data were calculated through 2^−∆∆Ct^ method.

### 2.4 Western blot

Cultured cardiomyocytes in each group were lysed with RIPA buffer, processed with BCA Protein Assay Kit (Beyotime, Shanghai, China), then treated with 12% SDS‐PAGE. Following transferring to the PDF membranes, the diluted primary antibodies against the internal control GAPDH and β‐MHC, BNP, ANF, as well as the HRP‐marked secondary antibodies were applied to probe with membranes. All antibodies were available from Abcam (Cambridge, MA). Protein band was observed using chemiluminescence (Pierce, Rockford, IL).

### 2.5 Immunofluorescence staining assay

Cardiomyocytes were fixed with 4% formaldehyde, permeated with 0.1% Triton X‐100 and cultured overnight with α‐actinin (Abcam) at 4℃. The appropriate secondary antibody was added for 1 hour, and cells were stained in DAPI solution for immunofluorescence capture using fluorescence microscope (Carl Zeiss Meditec, Oberkochen, Germany).

### 2.6 Plasmid transfection

The short hairpin RNAs (shRNAs) specifically targeted to PCDH17, SNHG14 or SP1 were constructed by GenePharma Company (Shanghai, China), with non‐specific shRNAs as control. To overexpress SNHG14 or PCDH17, the whole sequence of SNHG14 or PCDH17 was cloned into pcDNA3.1 vector (GenePharma), with empty vectors as control. The miRNA mimics/inhibitor for miR‐322‐5p or miR‐384‐5p and NC mimics/inhibitor were also acquired from GenePharma. Cardiomyocytes were transfected for 48 hours with Lipofectamine 2000 (Invitrogen).

### 2.7 Pull‐down assay

For RNA pull down, the lysates of cardiomyocytes obtained by using RIPA lysis buffer were incubated with the mixture of streptavidin agarose magnetic beads and biotin‐labelled probes of PCDH17 or miR‐322‐5p. Following 1 hour of incubation, the RNA‐protein complex was subjected to qRT‐PCR to examine RNA enrichment. For DNA pull down, the biotin‐labelled SNHG14 or PCDH17 promoter was mixed with beads and incubated with the cellular protein extracts all night. The non‐biotinylated promoters served as the negative control (NC). RNA enrichments were monitored using Western blot and qRT‐PCR.

### 2.8 RNA immunoprecipitation

Lysates of cardiomyocytes were incubated with the antibodies specific to human Ago2 or IgG (negative control) in RNA immunoprecipitation (RIP) buffer containing beads. At length, the immunoprecipitates were analysed by qRT‐PCR.

### 2.9 Luciferase reporter assay

The PCDH17 fragments covering the wild‐type and mutated miR‐322‐5p or miR‐384‐5p binding sites were inserted downstream of pmirGLO‐vectors (Promega, Madison, WI), termed PCDH17‐wt/mut. Recombinant reporter vectors SNHG14‐wt/mut were constructed similarly. Treated cardiomyocytes were co‐transfected with the above luciferase vectors and transfection plasmids for 48 hours, and then analysed using Luciferase Reporter Assay System (Promega). Besides, the SNHG14 or PCDH17 promoter with wild‐type and mutant SP1 binding sites were inserted to pGL3 vector (Promega) for promoter analysis.

### 2.10 Subcellular fraction assay

After washing in PBS, cardiomyocytes in lysis buffer were cultured on ice, centrifuged for obtaining cell cytoplasm. The supernatant was solubilized in cell disruption buffer, kept on ice for 20 minutes and centrifuged for 10 minutes. qRT‐PCR was employed to examine the SNHG14 expression in nucleus and cytoplasm.

### 2.11 FISH assay

Based on the protocols, FISH assay was conducted with the SNHG14‐FISH probes available from  RiboBio (Guangzhou, China) in hybridization buffer. Cell nuclei were processed in DAPI solution and monitored under the fluorescence microscope.

### 2.12 Statistical analyses

Statistical analyses were performed on Prism v.5.0 (GraphPad Software, La Jolla, CA), with significant level at *P* < .05. All measurement data of independent bio‐triplicates were exhibited with mean ± standard deviation (SD). Differences between groups were estimated employing Student's *t* test or one‐way analysis of variance.

## 3 RESULTS

### 3.1 PCDH17 is up‐regulated in CH in vivo and in vitro and its silence alleviates  Ang‐II induced CH

**Figure 1:**
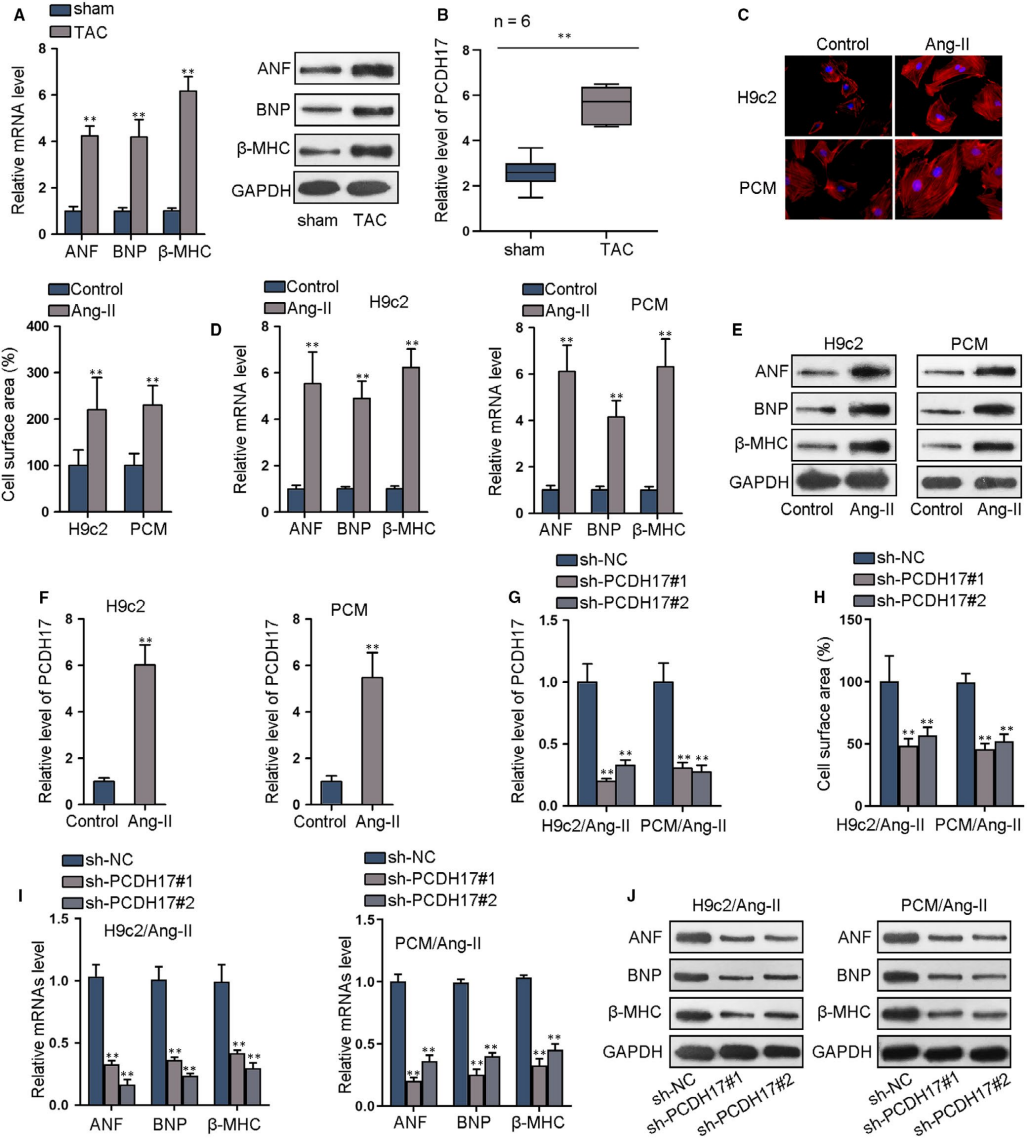
PCDH17 is up‐regulated in CH in vivo and in vitro and its silence alleviates Ang‐II‐induced CH. A, qRT‐PCR of mRNA level and Western blots of protein level of hypertrophic biomarkers TAC mouse heart versus sham control. B, qRT‐PCR of PCDH17 level in TAC mouse heart versus sham control. C, Immunofluorescence staining picture and quantification of cell surface area of Ang‐II‐induced cardiomyocytes (PCM and H9c2) versus control. D‐E, qRT‐PCR data and Western blots of the levels of hypertrophic biomarkers in cardiomyocytes under  Ang‐II treatment. F, qRT‐PCR detected PCDH17 expression in cardiomyocytes. G. The depletion efficiency of PCDH17 in Ang‐II‐induced cardiomyocytes was confirmed by qRT‐PCR. H, Immunofluorescence staining assay detected cell surface area of Ang‐II‐induced cardiomyocytes transfected with sh‐PCDH17#1/2. I‐J, qRT‐PCR and Western blot of levels of hypertrophic biomarkers in Ang‐II‐induced cardiomyocytes transfected with sh‐PCDH17#1/2. ^**^ *P* < .01

To test PCDH17 involvement in CH, its expression was evaluated in CH models in vivo and in vitro. In vivo model was established via transverse aortic constriction (TAC) in mice, and we illustrated the elevation of mRNA and protein levels of hypertrophic biomarkers (ANF, BNP and β‐MHC) in mouse heart of TAC group (Figure 1A and Figure S1A). Expectedly, PCDH17 level was elevated in TAC mouse heart versus sham mouse heart (Figure 1B). Primary cardiomyocytes (PCM) and H9c2 cells were applied to build in vitro model under Ang‐II treatment. Immunofluorescence staining assay revealed that cell surface area of H9c2 and PCM cells was significantly enlarged by treatment of Ang‐II (Figure 1C). Also, mRNA and protein of hypertrophic biomarkers were boosted in Ang‐II‐induced H9c2 and PCM cells (Figure 1D‐E and Figure S1B). PCDH17 level rose in PCM and H9c2 cells of  Ang‐II group versus control (Figure 1F). Based on these data, PCDH17 was suggested to participate in CH.

To demonstrate whether PCDH17 impacted CH in vitro, PCDH17 was knocked down and the knock‐down efficiency was verified in qRT‐PCR assay (Figure 1G). As a result, PCDH17 depletion reduced the cell surface area in Ang‐II‐induced PCM and H9c2 cells (Figure 1H). Further, qRT‐PCR and immunoblot confirmed the decline of hypertrophic biomarkers under PCDH17 depletion in Ang‐II‐induced PCM and H9c2 cells (Figure 1I‐J and Figure S1C). Thus, we concluded that PCDH17 was up‐regulated in CH and its knock‐down alleviated hypertrophic responses in Ang‐II‐induced cardiomyocytes.

### 3.2 MiR‐322‐5p and miR‐384‐5p target PCDH17

**Figure 2:**
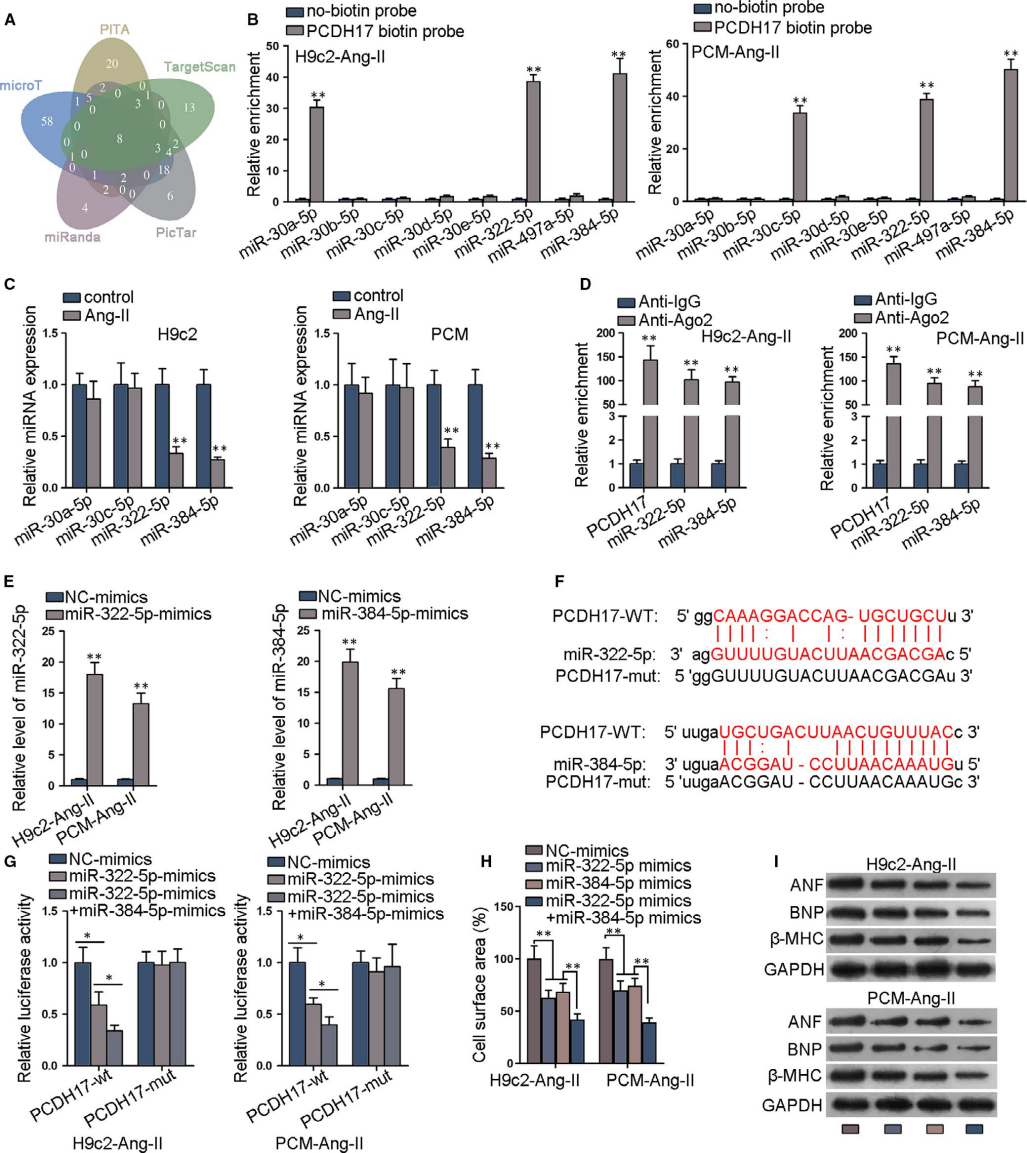
MiR‐322‐5p and miR‐384‐5p targets PCDH17. A, ENCORI predicted 8 miRNAs which bound to PCDH17. B, qRT‐PCR of the enrichment of 8 candidate miRNAs in the pull down of biotinylated PCDH17. C, qRT‐PCR data of levels of 4 candidate miRNAs in Ang‐II‐induced cardiomyocytes. D, RIP assay revealed relative enrichment of PCDH17, miR‐322‐5p and miR‐384‐5p in Ago2 and IgG group. E, qRT‐PCR verified overexpression efficiency of miR‐322‐5p and miR‐384‐5p. F, Binding sites on PCDH17 for miR‐322‐5p/miR‐384‐5p were predicted by ENCORI database. G, Luciferase activities of PCDH17‐wt/mut were quantified in cardiomyocytes under transfection of miR‐322‐5p mimics or miR‐322‐5p mimics + miR‐384‐5p mimics. H, Quantification of cell surface area in immunofluorescence assay in cardiomyocytes with suggested transfections. I, Western blots of hypertrophic markers in cardiomyocytes with suggested transfections. ^*^ *P* < .05, ^**^ *P* < .01

Subsequently, we sought to explain how PCDH17 was up‐regulated in CH. Since miRNAs are widely reported to directly bind with mRNAs and repress their expressions, we speculated that PCDH17 up‐regulation was attributed to the dysregulation of certain miRNAs in CH. Using ENCORI database,[^27^] we recognized 8 miRNAs combining the results of 5 prediction programs (microT, miRanda, PITA, PicTar, TargetScan), (Figure 2A). Among them, 4 miRNAs were verified to be pulled down by biotin‐labelled PCDH17 (Figure 2B), but only miR‐322‐5p and miR‐384‐5p levels were repressed by Ang‐II in PMC and H9c2 cells (Figure 2C). Consistently, down‐regulation of miR‐322‐5p and miR‐384‐5p was observed in TAC mouse heart versus sham control (Figure S2A). These data implied the association of miR‐322‐5p and miR‐384‐5p with PCDH17 and CH. Thereafter, we depicted the abundance of PCDH17, miR‐322‐5p and miR‐384‐5p in RIP products of Ago2 group but not in IgG group (Figure 2D). Further, overexpression efficiency of miR‐322‐5p and miR‐384‐5p in  Ang‐II treated PCM and H9c2 cells was verified by qRT‐PCR (Figure 2E). The binding sites in PCDH17 for miR‐322‐5p/miR‐384‐5p were predicted by ENCORI, and we mutated both sites in PCDH17 for the following luciferase reporter assay (Figure 2F). Overexpressing miR‐322‐5p alone declined luciferase activity of PCDH17‐wt, and such decline was strengthened by co‐overexpression of miR‐322‐5p/miR‐384‐5p, with luciferase activity of PCDH7‐mut unchanged (Figure 2G). Moreover, PCDH17 expression was slightly reduced by miR‐322‐5p overexpression and was remarkably reduced by overexpression of miR‐322‐5p/miR‐384‐5p (Figure S2B). Moreover, overexpressing miR‐322‐5p or miR‐384‐5p respectively reduced cell surface area and ANF, BNP, and β‐MHC proteins, and such impact was enhanced by jointly overexpressing miR‐322‐5p and miR‐384‐5p in PCM and H9c2 cells (Figure 2H‐I and Figure S2C). Based on these data, we concluded that PCDH17 was the target of miR‐322‐5p and miR‐384‐5p in CH.

### 3.3 SNHG14 up‐regulates PCDH17 expression via sponging miR‐322‐5p and miR‐384‐5p

**Figure 3:**
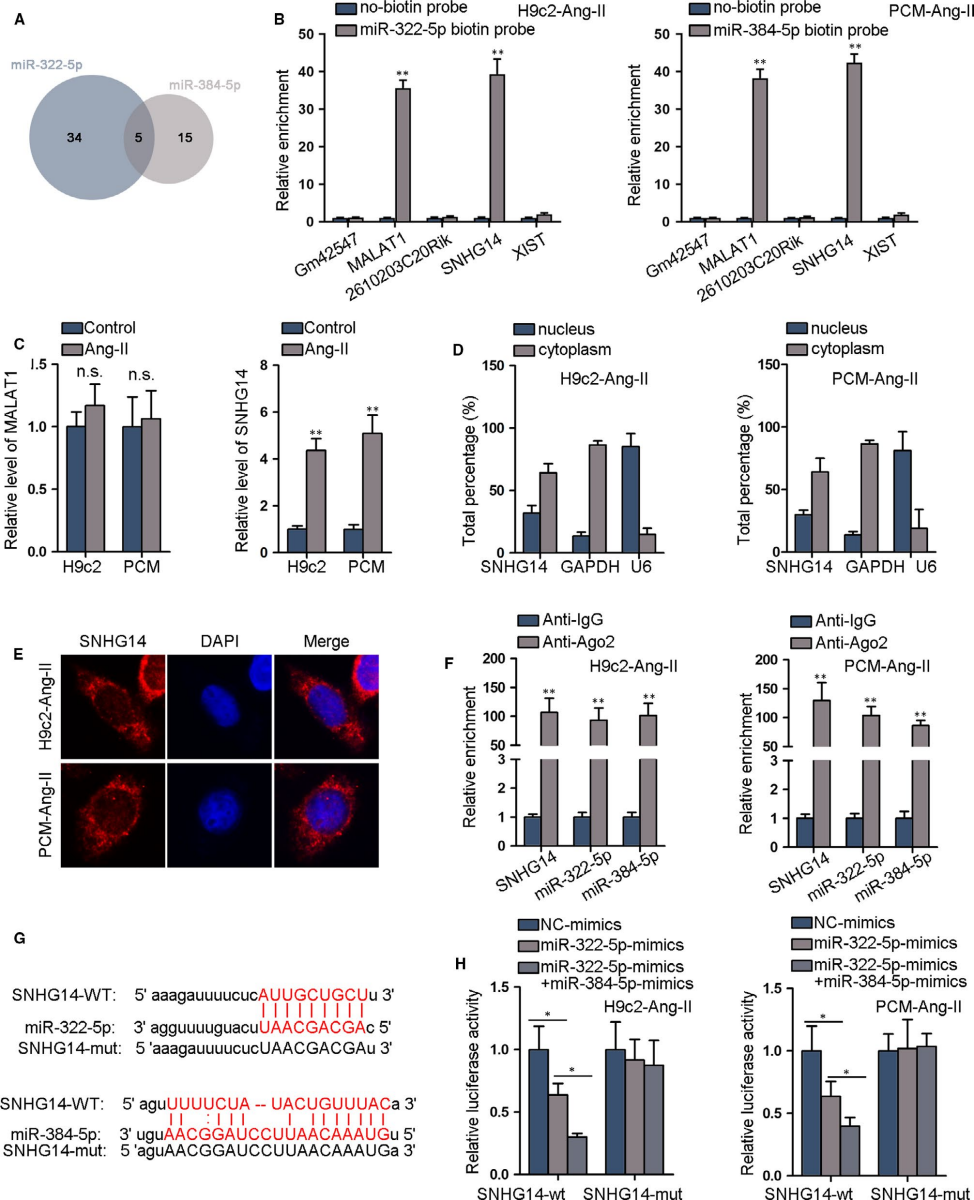
SNHG14 up‐regulates PCDH17 expression via sponging miR‐322‐5p and miR‐384‐5p. A, ENCORI predicted 5 lncRNAs which bound to both miR‐322‐5p and miR‐384‐5p. B, RNA Pull‐down assay revealed relative enrichment of 5 candidate lncRNAs pulled down by biotin‐labelled miR‐322‐5p and miR‐384‐5p. C, qRT‐PCR data of MALAT1 and SNHG14 expressions in cardiomyocytes under Ang‐II treatment. D‐E, Subcellular fraction quantification of SNHG14 expression in cytoplasm and nucleus and FISH staining image of SNHG14 localization in  Ang‐II treated cardiomyocytes. F, RIP assay of miR‐322‐5p, miR‐384‐5p and SNHG14 enrichment in IgG and Ago2 precipitates in cardiomyocytes. G, miR‐322‐5p/miR‐384‐5p binding sites in SNHG14 were predicted by ENCORI database. H, Luciferase activity of SNHG14‐wt/mut reporters was detected. ^*^ *P* < .05, ^**^ *P* < .01

LncRNAs are renowned as the miRNA sponge in ceRNA network, which contribute to the up‐regulation of genes in CH,[^28^, ^29^] so we explored the upstream lncRNAs for miR‐322‐5p and miR‐384‐5p. As was predicted by ENCORI, 5 lncRNAs were able to bind with both miR‐322‐5p and miR‐384‐5p (Figure 3A). RNA pull‐down results demonstrated that 2 of the 5 lncRNAs (MALAT1 and SNHG14) could be pulled down by biotin‐labelled miR‐322‐5p and miR‐384‐5p (Figure 3B), but only SNHG14 was significantly up‐regulated in Ang‐II‐induced cardiomyocytes (Figure 3C). SNHG14 level was also elevated in TAC mice compared with sham mice (Figure S3A). Thus, we paid attention to SNHG14. We conducted subcellular fraction assay and FISH assay to determine the subcellular location of SNHG14. The results revealed that SNHG14 was mainly distributed in the cytoplasm of Ang‐II‐induced cardiomyocytes (Figure 3D‐E). Subsequently, RIP assay revealed that SNHG14, miR‐322‐5p and miR‐384‐5p were highly enriched in Ago2 group but not in IgG group (Figure 3F). Moreover, the putative binding sites in SNHG14 for miR‐322‐5p/miR‐384‐5p and the mutated sites were demonstrated in Figure 3G. The luciferase activity of SNHG14‐wt was decreased by miR‐322‐5p mimics and such decrease was strengthened by miR‐322‐5p/miR‐384‐5p mimics, whereas luciferase activity of SNHG14‐mut was not impacted (Figure 3H). Besides, overexpressing neither miR‐322‐5p nor miR‐384‐5p affected SNHG14 expression, and co‐overexpressing miR‐322‐5p and miR‐384‐5p failed to alter SNHG14 level in  Ang‐II treated PCM and H9c2 cells as well (Figure S3B). Further, we knocked SNHG14 down and the knock‐down efficiency was verified by qRT‐PCR assay (Figure S3C). It was demonstrated that SNHG14 interference reduced PCDH17 expression (Figure S3D), suggesting that SNHG14 positively regulated PCDH17. Later, to further illustrated whether SNHG14 competitively binding to miR‐322‐5p and miR‐384‐5p to modulate PCDH17, we overexpressed wild‐type SNHG14 or SNHG14 (mut) (with both miR‐322‐5p and miR‐384‐5p sites mutated) in  Ang‐II treated PCM and H9c2 cells (Figure S3E). Shown in Figure S3F, the luciferase activity of PCDH17‐wt was suppressed by miR‐322‐5p/miR‐384‐5p overexpression, and overexpressing SNHG14 restored the luciferase activity. However, overexpressing SNHG14 (mut) failed to restore the decreased luciferase activity of PCDH17‐wt, with PCDH17‐mut activity unaltered all the way. Consistently, overexpression of SNHG14 rather than SNHG14 (mut) reversed the decline of PCDH17 mRNA and protein caused by miR‐322‐5p/miR‐384‐5p mimics in  Ang‐II treated PCM and H9c2 cells (Figure S3G‐H). According to these data, SNHG14 elevated PCDH17 expression via sponging miR‐322‐5p and miR‐384‐5p.

### 3.4 SNHG14 aggravates hypertrophic effects via sequestering miR‐322‐5p and miR‐384‐5p

**Figure 4:**
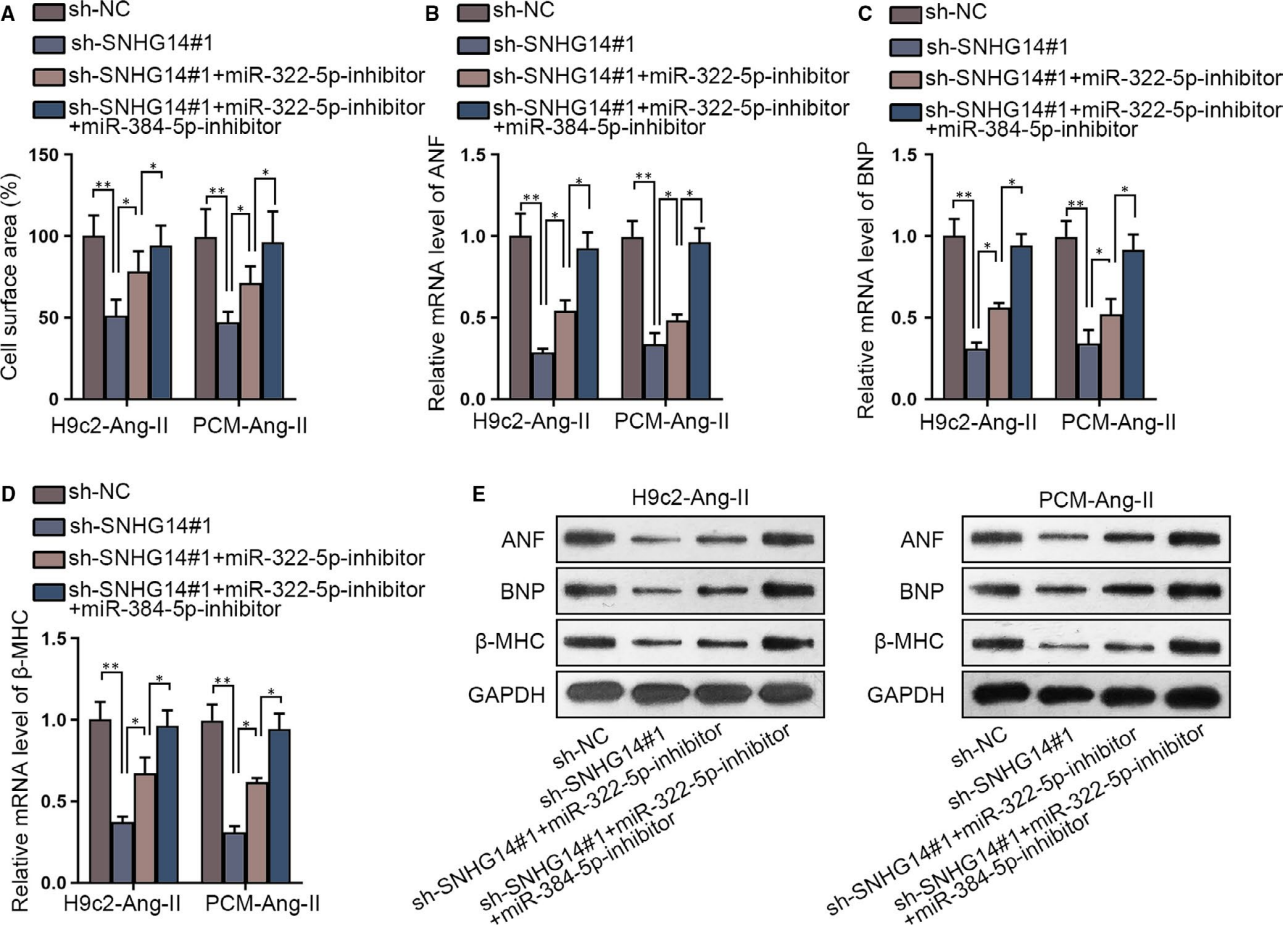
SNHG14 aggravates hypertrophic effects via miR‐322‐5p and miR‐384‐5p. A, Cell surface area of Ang‐II‐induced cardiomyocytes transfected with sh‐NC, sh‐SNHG14#1, sh‐SNHG14#1 + miR‐322‐5p‐inhibitor, sh‐SNHG14#1 + miR‐322‐5p/miR‐384‐5p‐inhibitor was revealed in immunofluorescence staining assay. B‐E, qRT‐PCR and Western blot were implemented to detect levels of hypertrophic biomarkers in Ang‐II‐induced cardiomyocytes in different groups. ^*^ *P* < .05, ^**^ *P* < .01

Thereafter, we went on to explore whether SNHG14 affected hypertrophic responses depending on miR‐322‐5p and miR‐384‐5p in Ang‐II‐induced cardiomyocytes. It was revealed that co‐transfection of miR‐322‐5p inhibitor partly restored cell surface area reduced by SNHG14 depletion, and a full restoration was obtained by co‐transfecting miR‐322‐5p/miR‐384‐5p inhibitors in Ang‐II‐induced cardiomyocytes (Figure 4A). Also, miR‐322‐5p inhibitor partly rescued mRNA and protein expressions of hypertrophic biomarkers repressed by SNHG14 depletion, and miR‐322‐5p/miR‐384‐5p inhibitor fully rescued the levels of the markers in Ang‐II‐induced cardiomyocytes (Figure 4B‐E and Figure S4A). Thus, we reached the conclusion that SNHG14 aggravated hypertrophic effects relying on miR‐322‐5p and miR‐384‐5p.

### 3.5 SP1 is the transcription factor for both SNHG14 and PCDH17

**Figure 5:**
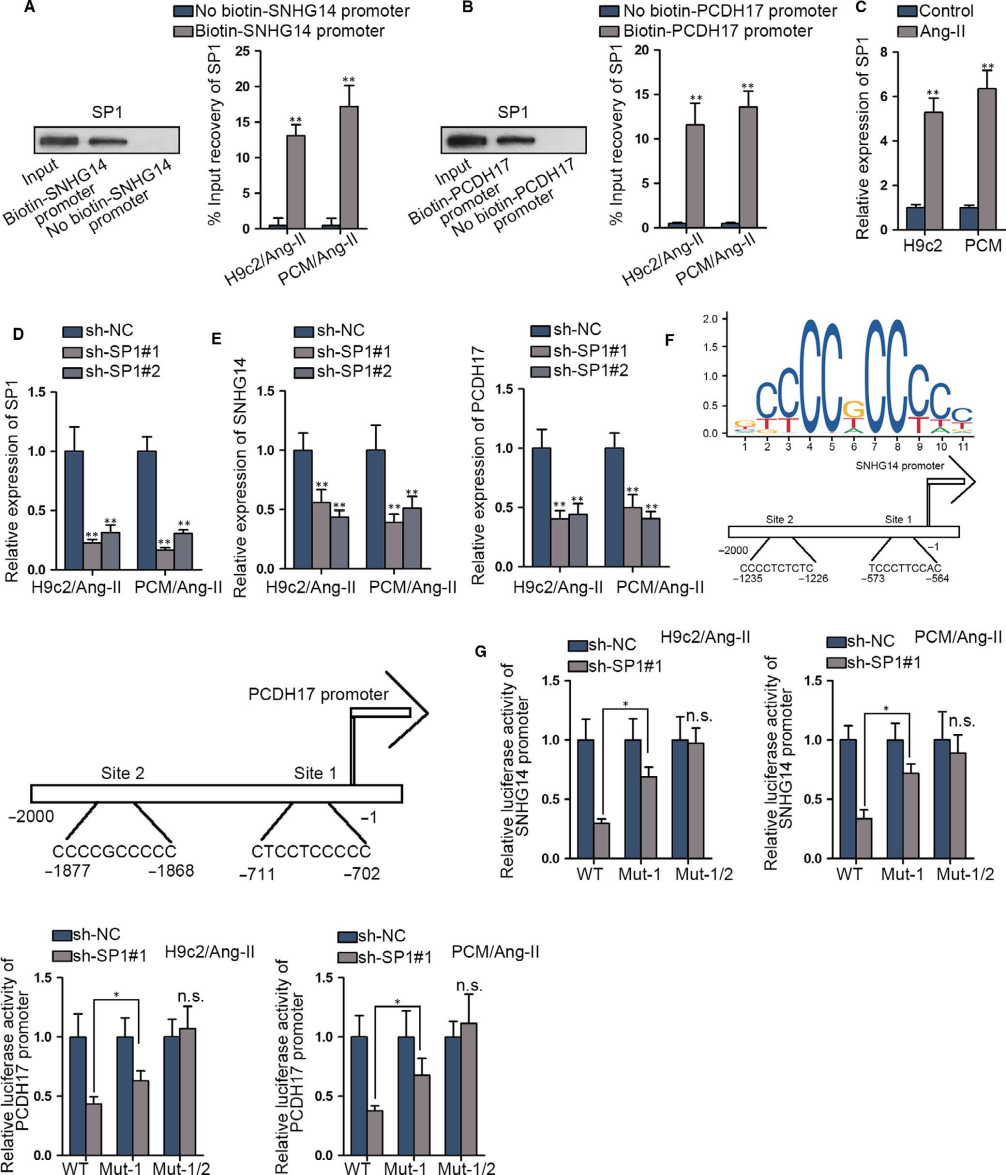
SP1 is the transcription factor for both SNHG14 and PCDH17. A‐B, DNA pull‐down assay verified between SP1 and PCDH17/SNHG14 promoter. C, qRT‐PCR detected relative SP1 expression in cardiomyocytes. D, qRT‐PCR verified SP1 knock‐down efficiency. E. qRT‐PCR detected expression of PCDH17/SNHG14 by SP1 knock‐down. F, DNA motif of SP1 predicted from JASPAR (http://jaspar.genereg.net/) and 2 predicted binding sites of SP1 in PCDH17/SNHG14 promoter. G, Luciferase activity detection verified the binding of SP1 to PCDH17/SNHG14 promoter. ^*^ *P* < .05, ^**^ *P* < .01. n.s. meant no significance

Since transcription factors are reported to induce the transcription of RNAs, we sought to identify the transcription factor for SNHG14 and PCDH17. As was demonstrated in DNA pull‐down assay, SP1 was significantly pulled down by biotin‐SNHG14 promoter and biotin‐PCDH17 promoter (Figure 5A‐B). Also, SP1 was overtly up‐regulated in Ang‐II‐induced cardiomyocytes as well as TAC mouse hearts (Figure 5C and Figure S4B). Then, we knocked SP1 down and the interference efficiency of SP1 was verified in qRT‐PCR assay (Figure 5D). SP1 depletion significantly down‐regulated SNHG14 and PCDH17 expressions in Ang‐II‐induced cardiomyocytes (Figure 5E). Next, the DNA motif of SP1 and the predicted SP1 sites on SNHG14 and PCDH17 promoters were illustrated in Figure 5F. Subsequently, luciferase activity of SNHG14 and PCDH17 promoter reporters declined under SP1 deficiency, and such decline was partly restored by mutating site 1 on SNHG14 and PCDH17 promoter, and fully restored by mutating both site 1 and site 2 (Figure 5G), verifying that SP1 bound to promoters of SNHG14 and PCDH17 at site 1 and 2. Based on these findings, we concluded that SP1 was the transcription activator for both SNHG14 and PCDH17.

### 3.6 SNHG14 and PCDH17 are required for the modulation of SP1 on hypertrophy in Ang‐II‐induced cardiomyocytes

**Figure 6:**
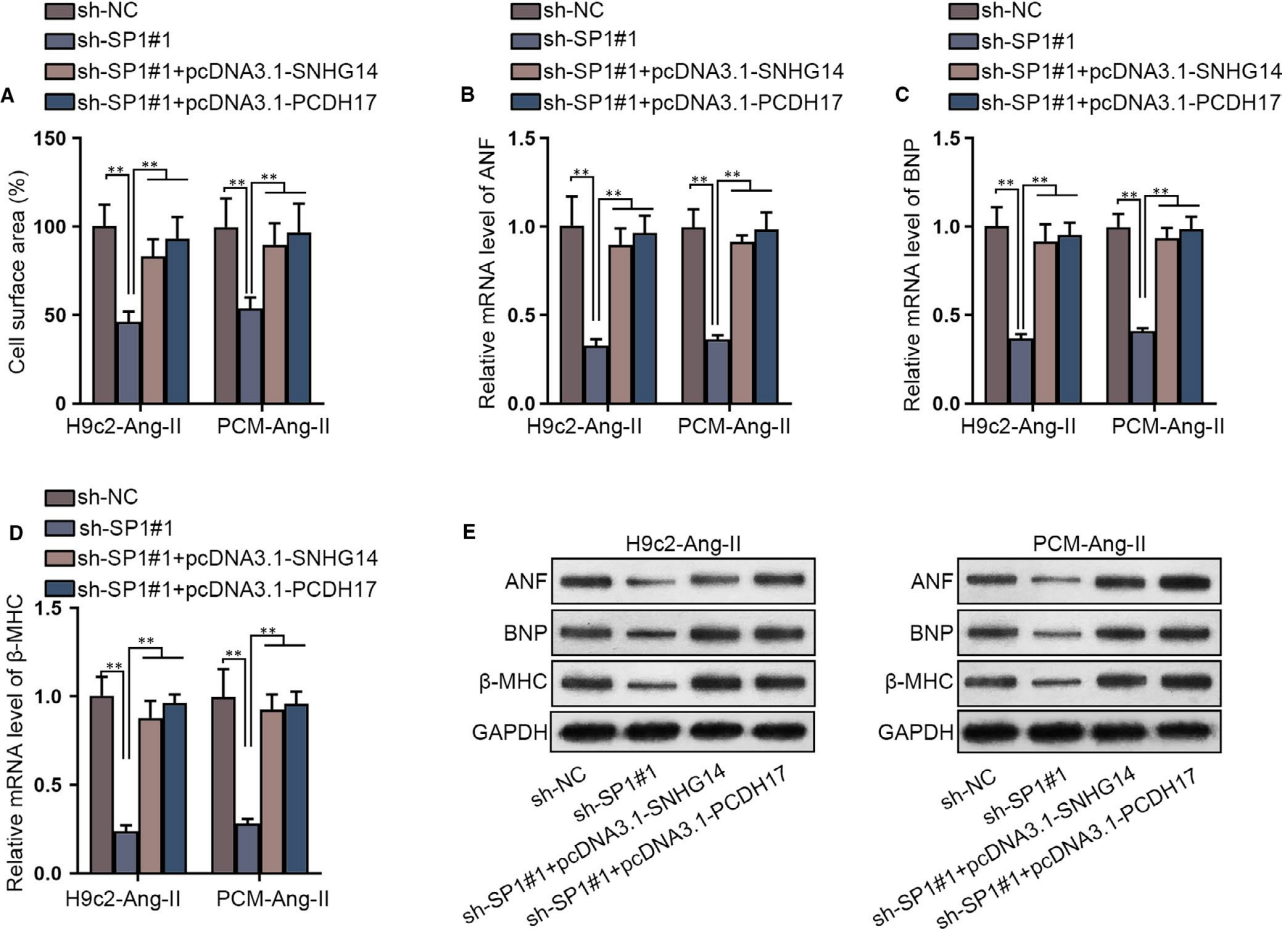
SP1 depletion mitigated pro‐hypertrophic effects of Ang‐II relying on SNHG14 and PCDH17 in cardiomyocytes. A, Cell surface area of Ang‐II‐induced cardiomyocytes in sh‐NC, sh‐SP1#1, sh‐SP1#1 + pcDNA3.1‐SNHG14 and sh‐SP1#1 + pcDNA3.1‐PCDH17 groups were revealed in immunofluorescence staining assay. B‐E, qRT‐PCR and Western blot detected the level of hypertrophic biomarkers in Ang‐II‐induced cardiomyocytes in different groups ^**^ *P* < .01

In this section, function of SP1 was verified. It was illustrated that SP1 depletion reduced cell surface area but co‐transfection of pcDNA3.1‐SNHG14 or pcDNA3.1‐PCDH17 impaired such effects in Ang‐II‐induced cardiomyocytes (Figure 6A). Also, SNHG14 depletion decreased mRNA and protein levels of hypertrophic biomarkers but co‐transfection of pcDNA3.1‐SNHG14 or pcDNA3.1‐PCDH17 recovered hypertrophic biomarker levels in Ang‐II‐induced cardiomyocytes (Figure 6B‐E and Figure S4C). Taken together, SP1 depletion abrogated pro‐hypertrophic effects of on cardiomyocytes relying on SNHG14 and PCDH17.

## 4 DISCUSSION

Cardiac hypertrophy (CH) is an adaptive reaction to pathological pressure overload and has association with heart failure or sudden cardiac death. Recently, the molecular functions in cardiac disorders have gradually been discovered. Former volumes reported that PCDH17 was up‐regulated in vascular smooth muscle cells of myocardial infarction,[^12^] and was verified to promote cardiomyocyte autophagy in diabetic cardiomyopathy,[^11^] indicating the relation between PCDH17 and heart disease. Our data first established the link between PCDH17 and CH by demonstrating that PCDH17 was up‐regulated in Ang‐II‐treated cardiomyocytes and in TAC mouse hearts. We demonstrated the function that PCDH17 depletion could alleviate Ang‐II‐induced hypertrophic responses in cardiomyocytes, suggesting PCDH17 as a positive regulator of CH in vitro.

Then, by starBase prediction, 8 miRNAs were identified to bind with PCDH17. Among them, only miR‐322‐5p and miR‐384‐5p were pulled down by PCDH17 biotin probe and were down‐regulated in Ang‐II‐treated cardiomyocytes. Previous reports have linked miR‐322‐5p to cardiac function. miR‐322‐5p expression is inhibited in heart of rats with pulmonary hypertension.[^30^] Also, miR‐322‐5p might possess higher potential for differentiation of cardiac resident stem/progenitor cells.[^31^] But miR‐384‐5p in heart disease has never been explored. Our study was the first to show down‐regulation of miR‐322‐5p and miR‐384‐5p in CH and reveal that overexpressing these 2 miRNAs reversed  Ang‐II induced CH in vitro.

Although it was widely reported that SNHG14 served as an oncogene in various cancers including hepatocellular carcinoma,[^19^] pancreatic cancer [^32^] and colorectal cancer,[^33^] the role of SNHG14 in cardiac disorders has not been illustrated. Herein, we first identified that SNHG14 was activated by  Ang‐II in cardiomyocytes and acted as a sponge for both miR‐322‐5p and miR‐384‐5p. SNHG14 depletion caused decreased cell surface area and hypertrophic biomarker levels in Ang‐II‐induced cardiomyocytes, and these effects are counteracted by down‐regulation of miR‐322‐5p and miR‐384‐5p, first suggesting the role of SNHG14/miR‐322‐5p/miR‐384‐5p axis in CH. Formerly, the ceRNA pattern in CH was widely seen in other reports.[^28^, ^34^, ^35^] Herein, we provided new data to confirm the competition between SNHG14 and PCDH17 for miR‐322‐5p and miR‐384‐5p in CH, uncovering the ceRNA network in which SNHG14 served as an endogenous sponge for miR‐322‐5p and miR‐384‐5p to up‐regulate PCDH17.

Moreover, we identified that SP1 was responsible for the transcription of both SNHG14 and PCDH17. SP1 served as a hypertrophic promoter in Ang‐II‐induced cardiomyocytes. Consistently, SP1 was previously reported to activate transcription of SNHG14 in clear cell renal cell carcinoma.[^36^] Also, SP1 plays the role of transcription factor for LINC00657,[^37^] lncRNA TINCR[^38^] and lncRNA POU3F3.[^39^] It was revealed that SP1 was an upstream activator for CTBP1‐AS2, which is a novel mediator in CH by recruiting FUS to stabilize TLR4.[^40^] SP1 serves as a transcription factor and has crucial interplays with miRNAs in pathological CH.[^41^] SP1 activates up‐regulation of lncRNA SYNE1‐AS1 and SYNE1‐AS1 positively regulated SP1 through sponging miR‐525‐5p in Ang‐II‐induced CH.[^42^] However, the relation of SP1 with SNHG14 and PCDH17 was first demonstrated by our data.

To conclude, present study uncovered a novel ceRNA mechanism of SNHG14/miR‐322‐5p/miR‐384‐5p/PCDH17 and additionally showed that SNHG14 and PCDH17 were both induced by SP1. SP1‐induced SNHG14 sponged miR‐322‐5p and miR‐384‐5p to elevate PCDH17 expression and to facilitate hypertrophic effects in Ang‐II‐induced cardiomyocytes, indicating SNHG14 as a putative biomarker for CH.

## CONFLICT OF INTEREST

The authors confirm that there are no conflicts of interest.

## AUTHORS’ CONTRIBUTION

Yadong Long involved in project administration, study design, data curation, data analysis, figures and wrote the original article. Lin Wang involved in investigation and preparation of the manuscript. Yadong Long and Zhiqiang Li involved in experiments; Zhiqiang Li involved in experiment record. All authors read and approved the final manuscript.
