# Supplementary figures and images for: SP1‐induced SNHG14 aggravates hypertrophic response in in vitro model of cardiac hypertrophy via up‐regulation of PCDH17
Source: J Cell Mol Med. 2020 May 21;24(13):7115–26. doi: 10.1111/jcmm.15073 (PMC7339172; doi:10.1111/jcmm.15073)

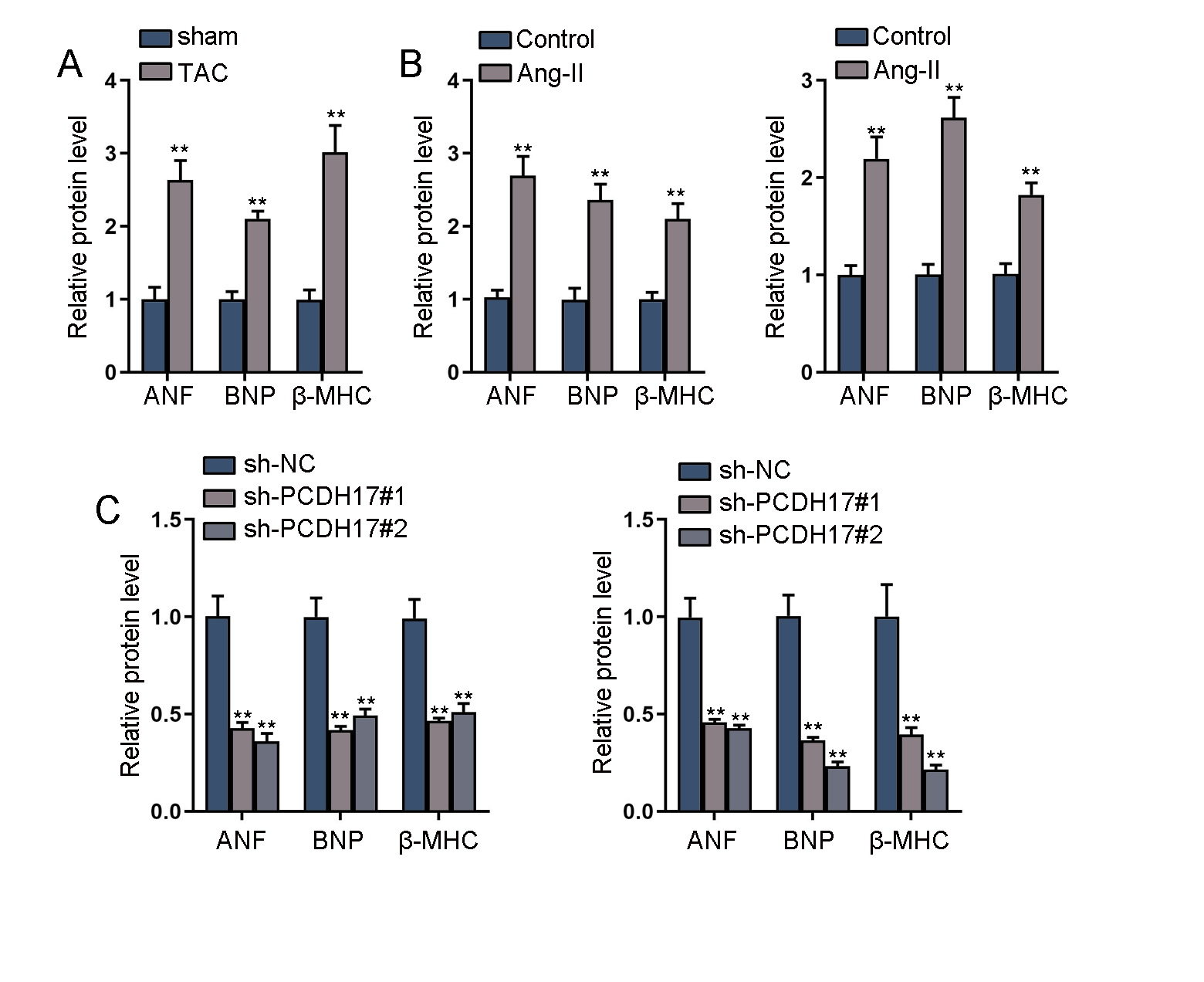

Supplement: Supplementary file 1 [file JCMM-24-7115-s001.tif]

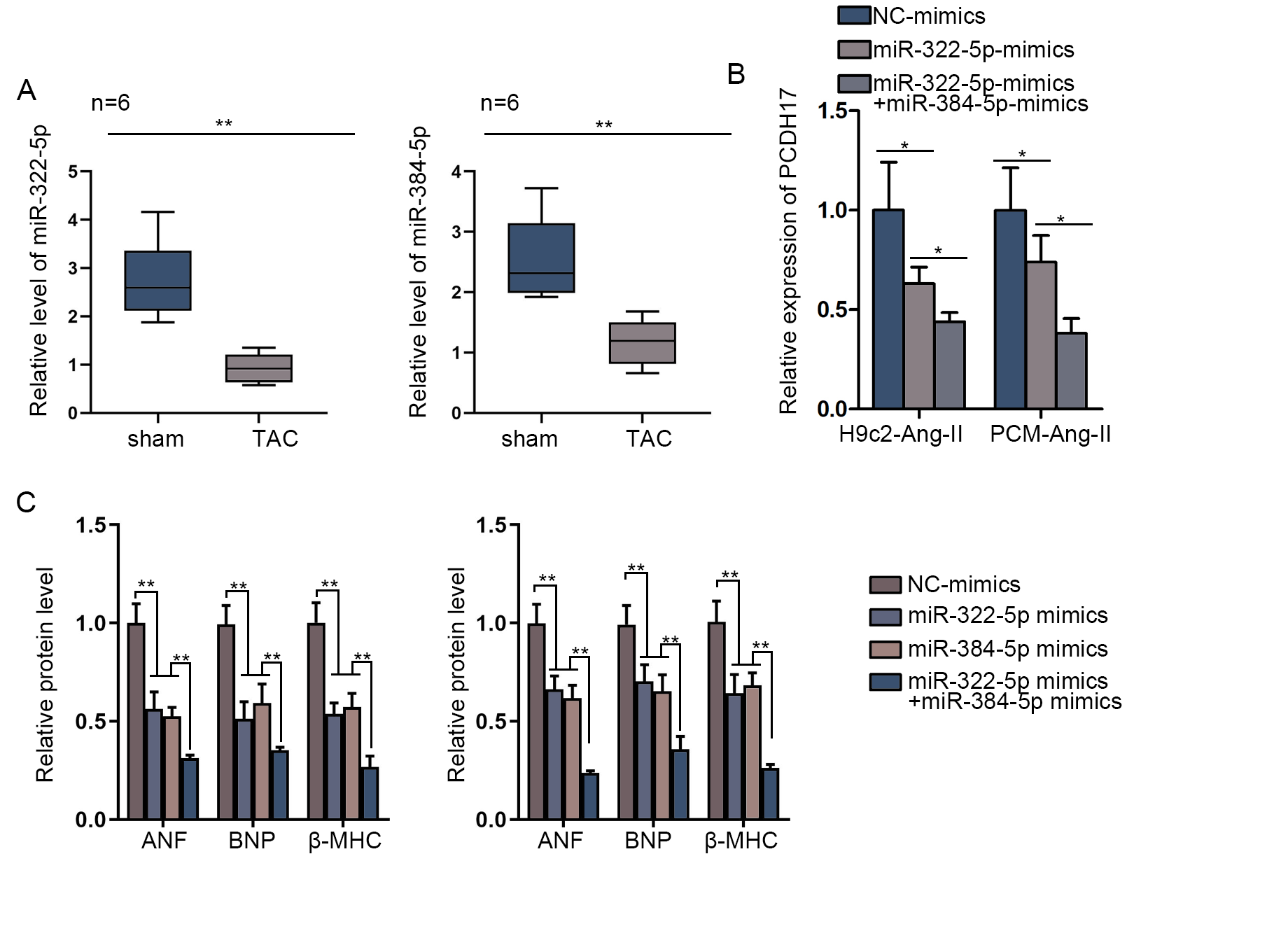

Supplement: Supplementary file 2 [file JCMM-24-7115-s002.tif]

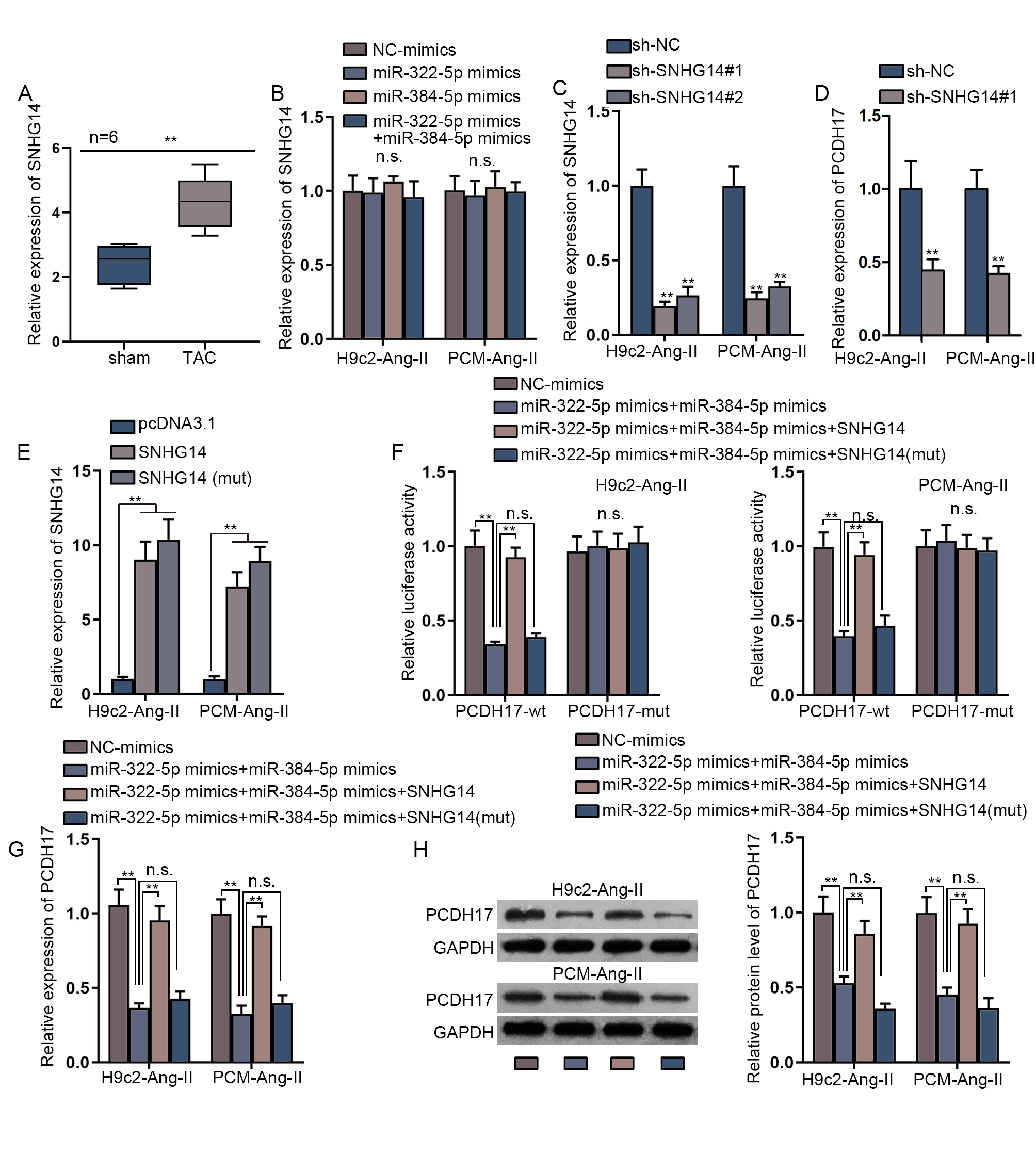

Supplement: Supplementary file 3 [file JCMM-24-7115-s003.tif]

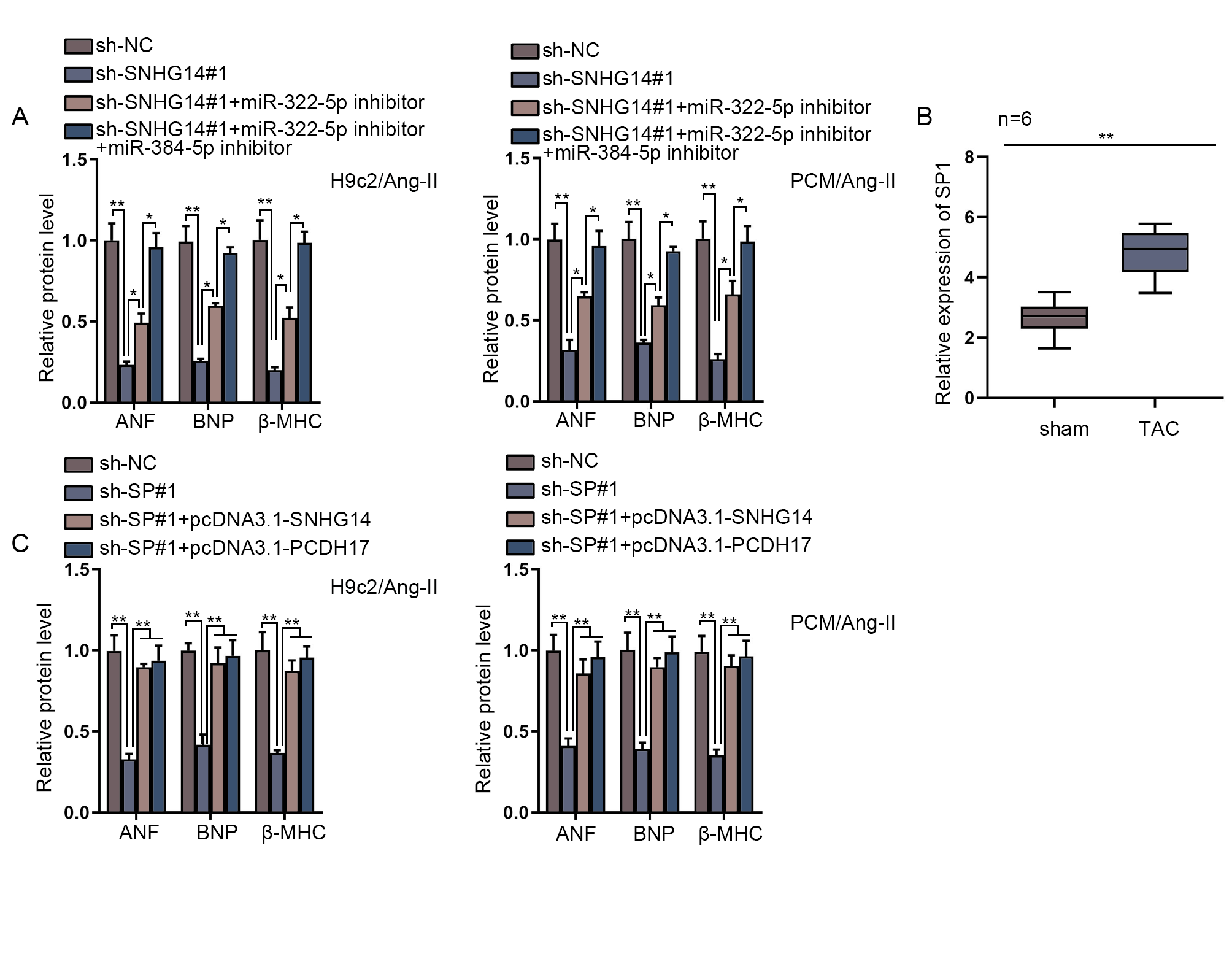

Supplement: Supplementary file 4 [file JCMM-24-7115-s004.tif]
